# Possible Mechanisms of Tau Spread and Toxicity in Alzheimer’s Disease

**DOI:** 10.3389/fcell.2021.707268

**Published:** 2021-07-28

**Authors:** Huiqin Zhang, Yu Cao, Lina Ma, Yun Wei, Hao Li

**Affiliations:** Institute of Geriatrics, Xiyuan Hospital, China Academy of Chinese Medical Sciences, Beijing, China

**Keywords:** tau, tauopathy, spread, toxicity, mechanism, Alzheimer’s disease

## Abstract

Tau is a protein that associates with microtubules (MTs) and promotes their assembly and stability. The protein loses its ability to bind MTs in tauopathies, and detached tau can misfold and induce the pathological changes that characterize Alzheimer’s disease (AD). A growing body of evidence indicates that tauopathies can spread between cells or connected regions. Pathological tau transmission in the brain of patients with AD and other tauopathies is due to the spread of various tau species along neuroanatomically connected regions in a “prion-like” manner. This complex process involves multiple steps of secretion, cellular uptake, transcellular transfer, and/or seeding, but the precise mechanisms of tau pathology propagation remain unclear. This review summarizes the current evidence on the nature of propagative tau species and the possible steps involved in the process of tau pathology spread, including detachment from MTs, degradations, and secretion, and discusses the different mechanisms underlying the spread of tau pathology.

## Introduction

The microtubule (MT)-binding protein tau is mainly expressed in the cytoplasm of neurons (Pérez et al., 2016) and plays key roles in regulating MT dynamics, axonal transport, and neurite outgrowth (Johnson and Stoothoff, 2004). Tau protein changes affect its MT-binding ability and consequently alter its normal physiological functions. For example, the phosphorylation of tau protein in and around its microtubule-binding domain (MBD) may neutralize its positive charges (Jho et al., 2010), alter MBD conformation, and lead to its detachment from MTs (Fischer et al., 2009). Once detached, tau accumulates in neurites and neuronal cell bodies, where it forms insoluble intracellular aggregates or inclusion bodies such as neurofibrillary tangles (NFTs), which are one of the major pathological features of Alzheimer’s disease (AD) (Lee et al., 2001; von Bergen et al., 2005; Zhang et al., 2009). Following detachment from MTs, tau can undergo structural transition, misfolding, and degradation (Frost et al., 2009). Tau can also be secreted into the extracellular space (Riemenschneider et al., 2003; Barthélemy et al., 2016) either in its naked form (Chai et al., 2012) or packaged in exosomes or other membranes (Saman et al., 2012; Simón et al., 2012; Polanco et al., 2016) following neuronal activity in mature neurons (Pooler et al., 2013; Dujardin et al., 2014b), neuron death (Gómez-Ramos et al., 2006), and/or when accumulated tau reaches a certain level in non-neuronal cells. In agreement with these findings, exogenous misfolded tau protein can be internalized by cells (Guo and Lee, 2011; Wu et al., 2013), a process that is mediated by heparin sulphate proteoglycans (HSPGs) and cell membrane receptors such as muscarinic (M1, M3) and a-amino-3-hydroxy-5-methyl-4-isoxazolepropionic acid (AMPA) receptors, as well as via endocytosis (Gómez-Ramos et al., 2008; Holmes et al., 2013; Tian H. et al., 2013). Once internalized, pathogenic misfolded tau proteins act as “seeds” that recruits soluble endogenous tau into larger aberrant conformations (Jucker and Walker, 2013) that slowly propagate across interconnected brain regions, as shown in various animal models (Clavaguera et al., 2009, 2013; Lasagna-Reeves et al., 2012). Fibrillar tau species can also transfer between cells and then recruit endogenous tau proteins onto their ends (Kfoury et al., 2012), a mechanism that may be responsible for the intracerebral spread of tau pathology (de Calignon et al., 2012; Iba et al., 2013).

Tau pathology spreading between neuronal cells and adjacent brain regions is a complex process involving many physiological and pathological aspects of tau protein, including its degradation, secretion, transmission, and toxicity. However, the exact mechanism underlying the spread of tau pathology after its release from cells remains unclear, and understanding these processes is the focus of an increasing number of studies (Le et al., 2012; Mohamed et al., 2013). There is some evidence that progressive accumulation of tau pathology in affected brain regions during AD development is due to the spread of aggregated tau along anatomically connected pathways (Hanger et al., 2014; Clavaguera et al., 2015; Lewis and Dickson, 2016). Accumulation of aggregates leads to neuronal loss and trans-synaptic spread of tau aggregates to more distal regions of the brain (Liu et al., 2012; Croft et al., 2017). The spread of extracellular species is the main pathway propagating neurofibrillary lesions and tau toxicity throughout different brain regions in neurodegenerative diseases (Iba et al., 2013; Pérez et al., 2018). A better understanding of the precise molecular mechanisms underlying tau propagation will contribute to the development of new therapeutic approaches for halting this process and provide new perspectives for the early diagnosis and prevention of tau pathologies (Fuster-Matanzo et al., 2018; Pérez et al., 2018). This review covers the most recent advances in our understanding of tau-spreading mechanisms, as well as the underlying implications of tauopathy-associated toxicity in AD. We further outline the possible mechanisms involved in pathology propagation including tau protein detachment from MTs; tau cleavage; tau degradation; and the release, uptake, and movement of pathogenic tau among synaptically connected neurons (Usenovic et al., 2015).

## Physiological Characteristics and Dissociation of Tau Protein From MTs

**FIGURE 1:**
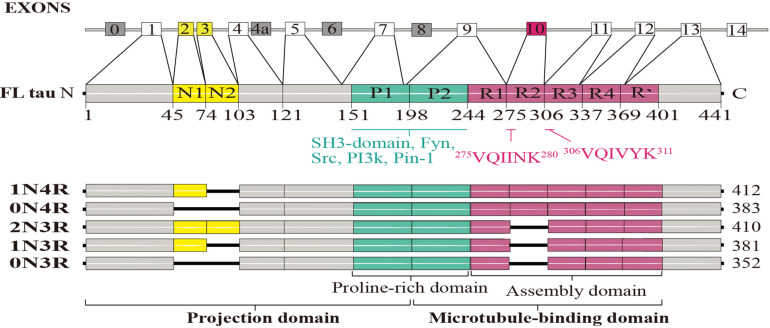
Structural features of the tau protein. The human tau gene, microtubule-associated protein tau (MAPT), contains 16 exons; alternative splicing of exons 2, 3, and 10 generates six tau protein isoforms: oN3R, 1N4R, 2N3R, 0N4R, 1N4R, and 2N4R. Full-length human tau protein (2N4R) contains 441 amino acids and four functional domains: an N-terminal projection region, a proline-rich domain, a microtubule-binding domain (MBD), and a C-terminal region. The N-terminal inserts are N1 and N2. The proline-rich domains P1 and P2 contain many phosphorylation sites and can bind to SH3 domains of other proteins, such as the tyrosine kinase Fyn. R1-R4 make up the repeat domain, which, together with the R’-flanking region, constitute the MT-binding domain. Two sequences are necessary for tau aggregation: ^275^VQIINK^280^ and ^306^VQIVYK^311^.

Tau protein can be divided into four functional domains: an N-terminal projection region, a proline-rich domain, an MBD, and a C-terminal region (Goedert and Spillantini, 2011). Tau can bind to the outside—and possibly also the inside—of MTs with the N- and C-terminal regions projecting out (Kar et al., 2003; Santarella et al., 2004). The N-terminal region can associate with the cell membrane and may be as a part of a membrane-associated complex; it also regulates the spacing between MTs (Maas et al., 2000; Al-Bassam et al., 2002). The proline-rich domain includes multiple phosphorylation sites (Augustinack et al., 2002) and can bind to Src homology 3 (SH3) domains of other proteins (Reynolds et al., 2008) such as the tyrosine kinase Fyn (Lee et al., 1998; Figure 1). Tau protein not only plays a crucial role in regulating MT dynamics but also promotes MT assembly and stabilization, processes that are required for morphogenesis and axonal transport in the nervous system (Johnson and Hartigan, 1999). However, the ability of tau to stabilize MTs is due in large part to its MBD (Gustke et al., 1994). Tau is thought to directly bind MTs through positively charged tandem repeat sequences within its MBD that are attracted to tubulin’s negatively charged residues (Kar et al., 2003; Jho et al., 2010).

The human tau gene, microtubule-associated protein tau (MAPT), is located on chromosome 17q21 and comprises 16 exons. Alternative splicing of exons 2, 3, and 10 generates six isoforms of the tau protein (Goedert et al., 1989). They are equally expressed in central nervous system neurons of a healthy adult brain (Goedert et al., 1989; Garcia and Cleveland, 2001), and can be grouped into tau-3R class members, which contain three MT-binding repeats (MTBRs), and tau-4R class members contain four MTBRs (Figure 1). Because of the extra repeat, 4R isoforms have a higher affinity for MTs and can therefore bind and stabilize MTs more efficiently (Goedert and Spillantini, 2011; Morris et al., 2011; Chen and Jiang, 2019).

**FIGURE 2:**
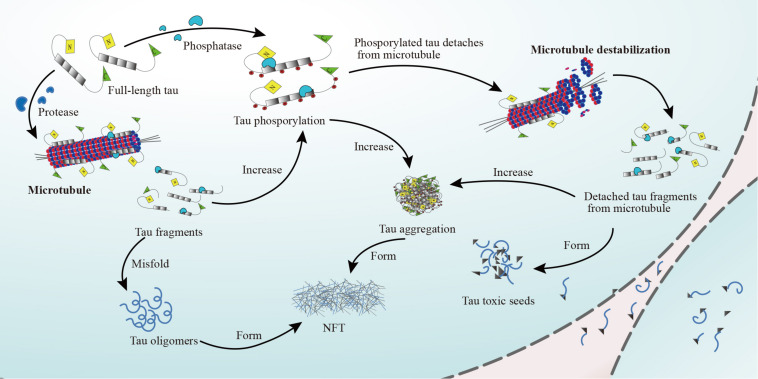
Detachment of tau protein from MTs. The full-length tau protein binds to MTs, stabilizing them under the regulation of phosphatases (e.g., PP1, PP2A, PP2B, PP2C, and PP5). When tau protein phosphorylates, tau protein will detach from MTs. The microtubules will destabilize and dissociate. Detached tau fragments form tau aggregation, which ultimately leads to the formation of NFTs. At the same time, phosphorylated tau protein and detached tau fragments misfold and form tau oligomers, which are the precursors of NFTs, or toxic tau seeds.

Tau protein functions are regulated by complex post-translational modifications including phosphorylation, glycation, isomerization, sumoylation, nitration, acetylation, and truncation (Morris et al., 2011). Moreover, tau contains numerous serine and threonine residues, so almost 20% of the protein has the potential to be phosphorylated (Wang and Mandelkow, 2016). The phosphorylation state of tau and its MT-binding affinity are controlled by a balance between kinase and phosphatase activity (Brandt et al., 1995; Shackelford and Yeh, 1998). Tau phosphorylation is mediated by MT affinity-regulating kinases (also known as PAR1 kinases), cyclic AMP-dependent protein kinase A, calcium (Ca^2+^), or calmodulin-dependent protein kinase II (CaMKII), and tyrosine kinases like Src family members (Hanger et al., 2009). The activation of tau phosphorylation-associated kinases (e.g., CDK-5 and GSK-3β) can induce tau hyperphosphorylation, which drives dissociation of tau protein from MTs (Hanger et al., 2009). Dissociated tau can misfold and become toxic seeds that are secreted from the cell. In contrast, fully dephosphorylated tau binds to MTs with high affinity (Shackelford and Yeh, 1998). Tau dephosphorylation is mediated by protein phosphatases 1, 2A, 2B, 2C, and 5 (Hanger et al., 2009; Pérez et al., 2018). In addition, detached tau can accumulate in neurites and neuronal cell bodies, first forming insoluble filaments and eventually NFTs (Lee et al., 2001; von Bergen et al., 2005; Figure 2). Abnormal tau phosphorylation decreases MT binding and likely increases tau-tau interactions (Morris et al., 2011). Physiologically, tau is continuously phosphorylated and dephosphorylated to ensure its proper function; however, when the balance shifts toward phosphorylation, tau affinity for MTs decreases (Alonso et al., 1997), resulting in higher cytosolic tau levels, which facilitates tau aggregation (Wegmann et al., 2018). Additionally, aberrantly phosphorylated tau protein appears to sequester other microtubule-associated proteins (MAPs), further destabilizing MTs (Alonso et al., 1997). Similarly, aberrant tau phosphorylation and self-aggregation lead to the formation of oligomers and higher-order aggregates that can lead to tau detachment from MTs and disturb the binding of other MAPs to MTs (von Bergen et al., 2000).

## Tau Protein Cleavage and Degradation

Tau cleavage and degradation are closely related to its pathological transmission and aggregation. The cleavage of tau generates seeds that promote tau aggregation (Wang et al., 2014), alter tau clearance, and can impair cognition and motor ability (Bondulich et al., 2016). Tau cleavage occurs at its N- and C-terminals (García-Sierra et al., 2008) and depends on associated proteases, mainly caspase-3, calpain, and cathepsin L (Wang et al., 2009; de Calignon et al., 2010; Table 1). Tau truncation can be initiated by caspase-3, which cleaves tau at residue D421, its predominant target (Chung et al., 2001). Caspase-3 can be activated by amyloid-beta (Aβ) and caspase-2 (Gamblin et al., 2003), following which caspase-3 can cleave tau at D25-Q26 (Corsetti et al., 2008) and D421-S422 (Gamblin et al., 2003). Cleavage at D421-S422 produces the N-terminal fragment (NTF) tau_1–421_ (Tau-C) (Nicholls et al., 2017). Caspase-2 is a protease that initiates activation of other caspases; it cleaves tau at D314-L315 to produce a soluble, toxic NTF (tau_1–314_; △tau_314_; Zhao et al., 2016). Tau_1–314_ levels were elevated in the brains of mice with mild cognitive impairment (MCI) and in the brains of AD patients compared with healthy controls (Zhao et al., 2016). Moreover, memory deficits were ameliorated following the application of anticaspase-2 morpholino oligonucleotides (Zhao et al., 2016). However, *in vitro* experiments using recombinant tau preparations suggested that caspase-2 preferentially cleaves tau at D421-S422 (Zhao et al., 2016). Another effector caspase, caspase-6, and puromycin-sensitive aminopeptidase (PSA) were reported to cleave recombinant human tau_441_ at D13-H14 (Sengupta et al., 2006), which was sufficient to cause axonal degeneration (Sokolowski et al., 2014). Caspase-6 cleavage at D402-T403 produces the NTF tau_1–402_, a cerebrospinal fluid (CSF) biomarker for AD (Ramcharitar et al., 2013). In addition, calpain-1 and -2 can both cleave tau (Chesser et al., 2013) and play opposing roles in regulating synaptic plasticity and promoting neurodegeneration (Baudry and Bi, 2016). The 17-kDa tau_45–230_ fragment is generated through cleavage by calpain-1 at K44-E45 (Yang and Ksiezak-Reding, 1995) or via calpain-1 (Park and Ferreira, 2005) or -2 (Garg et al., 2011) action at R230-T231. Tau is also cleaved by calpain-1 at R242-L243 to produce the 24-kDa C-terminal fragment (CTF) tau_243–441_ (Matsumoto et al., 2015). The levels of the tau_243–441_ fragment increase with aging in a tauopathy mouse model (Tg601 mice expressing wild-type human tau), and CTFs with sizes ranging from 20 to 28 kDa are present in brain samples from patients with AD and familial frontotemporal dementia (Matsumoto et al., 2015). Tau_243–441_ can proficiently propagate to other tau-expressing cells, leading to further seeding and tau_441_ phosphorylation (Matsumoto et al., 2015). Interestingly, tau_441_ build up can activate calpain-2, which leads to the degradation of nicotinic acetylcholine receptor subunit 4 (Yin et al., 2016), a crucial component of cholinergic signaling. Calpain-2 activation by tau_441_ creates a positive feedback loop that enhances neurotoxic tau fragment generation. Cathepsins B, D, and L can also proteolytically cleave tau. One study reported that cathepsin B was associated with intracellular NFTs, and its expression was elevated around Aβ plaques (Ii et al., 1993). Cathepsin D can cleave recombinant tau at F8-E9, M419-V420, and L436-A437; there is another potential cleavage site at either T427-L428 or L428-A429 and additional cleavage sites in D34-G161, P200-K257, and K267-D358 (Kenessey et al., 1997). In Neuro-2A murine cells, cathepsin L can cleave tau_244–372_ (lacking K280; tau_RD_△K), which is a mutated version of the aggregation-prone MBD tau fragment (García-Sierra et al., 2008). Tau_RD_△K cleavage by cathepsin L depends on an initial cleavage at K257-S258 by an unknown cytosolic protease that generates tau_258–372_; this fragment is further cleaved by cathepsin L at V363-P364 to produce tau_258–363_ that is subsequently cleaved at I360-T361 to produce tau_258–360_ (García-Sierra et al., 2008). Tau_258–360_ and tau_258–363_ induce aggregation of intact tau_RD_△K and full-length tau, which is coincident with lysosomal leakage (García-Sierra et al., 2008). Asparagine endopeptidase-mediated tau cleavage occurs at both N255-V256 and N368-K369 and produces five tau fragments. Among them, tau_1–368_ and tau_256–368_ were the only critical drivers of enhanced apoptosis in rat primary neurons, while only tau_1–368_ has been found in the brains of patients with AD (Zhang et al., 2014; Quinn et al., 2018).

**TABLE 1:** Tau protein cleavage at different sites by known proteases.

Protease	Cleavage site	Cleavage domain	Tau fragment	Effect on AD	References
Caspase-2	D314-L315	MBD	Tau_1_-_314_ (△tau_314_)	Caspase-2 preferentially cleaves recombinant tau at D421-S422 compared to D314-L315. Detached from MTs, tau invades healthy dendritic spines. This impaired synaptic transmission and drove hippocampal neuronal loss, but spatial memory deficits and toxicity were only observed when tau_1–314_ promoted mislocalization of full-length tau to dendritic spines.	Zhao et al., 2016
			Tau_315–441_	Unclear	Zhao et al., 2016
Caspase-3	D421-S422	C-terminus	Tau_1–421_ (Tau-C)	Linked to other tauopathies	Gamblin et al., 2003; Corsetti et al., 2008; Zhao et al., 2015
	D25-Q26	N-terminus	Tau_1–25_	No toxicity to neurons	Corsetti et al., 2008
Caspase-3 Calpain-1	D25-Q26 K44-E45	N-terminus	Tau_26–44_	Caused NMDAR-mediated cell death in rat CGCs	Gamblin et al., 2003; Park and Ferreira, 2005; Corsetti et al., 2008; Garg et al., 2011; Zhao et al., 2015
Caspase-3 Calpain-1 Calpain-2	D25-Q26	N-terminus	Tau_26–230_ (20–22kDa fragment)	Enriched in synaptic mitochondria; binds to Aβ peptides and exacerbates mitochondrial dysfunction. Induced NMDAR-mediated death of rat CGCs.	Gamblin et al., 2003; Park and Ferreira, 2005; Corsetti et al., 2008; Garg et al., 2011; Zhao et al., 2015
	R230-T231	MBD			
Caspase-6	D13-H14	N-terminus	Tau_1–13_	Caused axonal degeneration	Sengupta et al., 2006; Sokolowski et al., 2014
			Tau_14–441_	Possible role in tangle maturation	Sengupta et al., 2006; Sokolowski et al., 2014
	D402-T403	C-terminus	Tau_1–402_ (Tau△Casp6)	Serves as a CSF biomarker of neurodegeneration in AD	Ramcharitar et al., 2013
			Tau_403–441_	Unclear	Ramcharitar et al., 2013
Caspase-1, -3, -6, -7, -8	D421-S422	C-terminus	Tau_422–441_	Unclear	Quinn et al., 2018
	D421-S422	C-terminus	Tau_151–421_ (△tau)	Led to tau aggregation and disrupted axonal transport, mitochondrial function, Golgi apparatus, and synaptic protein levels	
	K150-I151	N-terminus			
PSA	D13-H14	N-terminus	Tau_1–13_	Caused axonal degeneration	Sengupta et al., 2006; Sokolowski et al., 2014
Calpain-1	K44-E45	N-terminus	Tau_1–44_	Caused NMDAR-mediated cell death in rat CGCs	Park and Ferreira, 2005; Garg et al., 2011
			Tau_45–441_	Unclear	Yang and Ksiezak-Reding, 1995
	R242-L243	MBD	Tau_243–441_ (24kDa CTF)	Accelerated the propagation to other tau-expressing cells, causing further seeding of aggregates and tau_441_ phosphorylation; reduced capacity for promoting MT assembly compared with tau_441_	Matsumoto et al., 2015
			Tau_1–242_	Unclear	Matsumoto et al., 2015
Calpain-1 and thrombin, Calpain-1 and -2	K44-E45	N-terminus	Tau_45–230_ (17kDa fragment)	Caused synapse loss and behavioral abnormalities; impaired organelle transport	Yang and Ksiezak-Reding, 1995; Park and Ferreira, 2005; Garg et al., 2011; Quinn et al., 2018
	R230-T231	MBD			
Calpain-2 and thrombin, Calpain-1 and -2	A2-E3	N-terminus	Tau_3–230_	Unclear	Quinn et al., 2018
	R230-T231	MBD			
	R230-T231	MBD	Tau_125–230_	Not toxic	
	Q124-A125	Projection domain			
Calpain-2	A2-E3	N-terminus	Tau_3–124_	Unclear	Quinn et al., 2018
	Q124-A125	Projection domain			
Cathepsin L	K257-S258	MBD	Tau_258–372_	Unclear	García-Sierra et al., 2008
	V363-P364	MBD	Tau_258–363_	Induced the aggregation of full-length tau and intact tau_RD_△K coincident with lysosomal leakage	
	I360-T361	MBD	Tau_258–360_		
AEP	N255-V256	MBD	Tau_1–255_	Unable to promote MT polymerization or aggregation into PHFs, but tau_1–255_ had strong AT8 immunoreactivity (at phosphorylation sites S202 and T205)	Zhang et al., 2014; Quinn et al., 2018
	N255-V256	MBD	Tau_256–441_	Significantly reduced MT polymerization and showed increased propensity to aggregate into PHFs compared with tau_441_	
	N255-V256	MBD	Tau_1–368_, Tau_256–368_	Enhanced apoptosis; increased ability to aggregate into PHFs	
	N368-K369	MBD			
	N368-K369	MBD	Tau_369–441_	Unable to cause MT polymerization and aggregation into PHFs	
Thrombin	R155-G156	Proline-rich domain	Tau_156–441_	Unclear	Quinn et al., 2018
	R155-G156	Proline-rich domain	Tau_156–209_	Unclear	
	R209-S210	MBD			
	R209-S210	MBD	Tau_210–441_	Unclear	
	R209-S210	MBD	Tau_210–230_	Unclear	
	R230-T231	MBD			
	R230-T231	MBD	Tau_231–441_	Unclear	
Chymotrypsin	Y197-S198	Projection domain	Tau_1–197_	Unclear	Quinn et al., 2018
	Y197-S198	MBD	Tau_198–441_	Unclear	
ADAM10	A152-T153	Proline-rich domain	Tau_1–152_	Unclear	Quinn et al., 2018
			Tau_153–441_ (Tau-A)	Unclear	

*Aβ, amyloid-beta; AD, Alzheimer’s disease; ADAM10, a disintegrin and metalloprotease 10; AEP, asparagine endopeptidase; AMPA, a-amino-3-hydroxy-5-methyl-4-isoxazolepropionic acid; CGC, cerebellar granule cell; CSF, cerebrospinal fluid; CTF, C-terminal fragment; MBD, microtubule-binding domain; MT, microtubule; NFT, neurofibrillary tangle; NMDAR, N-methyl-D-aspartate receptor; NTF, N-terminal fragment; PHF, paired-helical filament; PSA, puromycin-sensitive aminopeptidase; PSP, progressive supranuclear palsy.*

Intracellular tau degradation mainly involves two major proteolytic systems: ubiquitin-proteasome and autophagy-lysosomal (Chesser et al., 2013; Lee et al., 2013; Guo et al., 2017). Full-length tau is cleared via the former system (Liu et al., 2009; Dolan and Johnson, 2010), whereas its mutated and truncated forms appear to be degraded through the latter pathway (García-Sierra et al., 2008; Fernández-Montoya and Pérez, 2015). Moreover, tau phosphorylation can exacerbate its proteolytic degradation (Kenessey et al., 1997), while hyperphosphorylation is associated with impaired tau degradation via the ubiquitin-proteasome system (Dickey et al., 2007) and tau secretion. Tau can undergo natural self-degradation at cysteine residues by acetyl-coenzyme A-induced autoacetylation (Cohen et al., 2016) following its dissociation from MTs (Cohen et al., 2013, 2016). Tau accumulation can also result from increased expression or decreased degradation of the protein (Barton et al., 1990; Zhang et al., 2014), and degradation is impaired by a modified form of tau (Zhang et al., 2014). Thus, tau acetylation may both inhibit and facilitate its degradation and also suppress its phosphorylation and aggregation (Min et al., 2010; Cook et al., 2014). Acetylated tau has been found in brains from patients with AD and other tauopathies. For example, Lys174 acetylation was recently described in AD brains and may be a critical determinant for tau-induced toxicity by delaying tau turnover (Min et al., 2015). This result indicates that targeting tau acetylation could be a novel therapeutic option for AD and other human tauopathies.

## Secretion and Release of Tau Fragments

**FIGURE 3:**
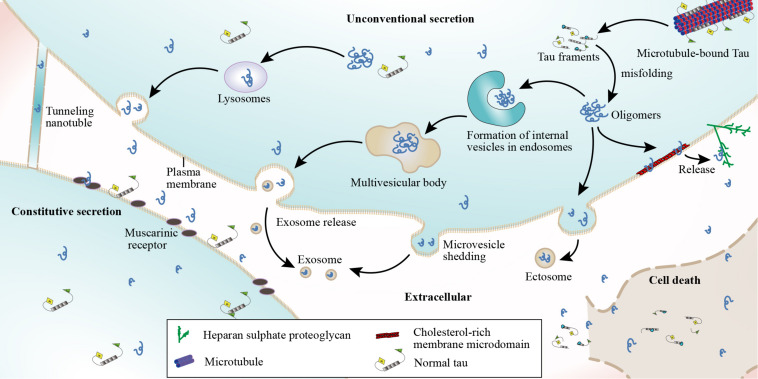
Tau protein secretion and release. The possible mechanisms underlying the presence of tau in the extracellular space include cell death, neuroactive stimulation, constitutive secretion, and unconventional secretion. A small amount of tau can be released into the extracellular space in free form or by penetrating adjacent membranes, which can be achieved by stimulating neuroactivity or constitutive secretion. However, the methods involved in unconventional tau protein secretion include (1) non-vesicular direct translocation across the plasma membrane from the cytoplasm and extracellular trapping via HSPGs, (2) release via secretory lysosomes that fuse with the plasma membrane and release their contents into the extracellular space, (3) microvesicle shedding from the plasma membrane, and (4) vesicle-mediated exosome release. Tau can also be secreted by both neuronal and non-neuronal cells when tau protein is exogenously expressed, while extracellular tau can bind muscarinic (M1, M3) receptors. Tau may also be released through nanotubes that connect the cytoplasmic compartments of adjoining cells.

Although tau is intracellular, recent studies have indicated that it is also present in the extracellular space both *in vitro* and *in vivo* (Kim et al., 2010a,b; Chai et al., 2012). Tau has been detected in both the CSF and interstitial fluid of tau transgenic mouse brains (Yamada et al., 2011; Barten et al., 2012). *In vitro* studies have shown that human tau is secreted by both neuronal and non-neuronal cell lines when the protein is overexpressed (Chai et al., 2012; Saman et al., 2012; Simón et al., 2012; Pérez et al., 2016). Extracellular tau may elicit toxicity (Gómez-Ramos et al., 2006; Díaz-Hernández et al., 2010) by binding to cellular receptors such as muscarinic receptors (Gómez-Ramos et al., 2008), but the mechanisms by which tau exits into the extracellular space remain unclear (Nickel and Rabouille, 2009). Another study showed that tau can be released into the extracellular space following neuronal death (Simón et al., 2012) and can subsequently be identified in the CSF (Iqbal et al., 2005). Secreted extracellular tau can be toxic to surrounding cells through interactions with specific cell receptors (Gómez-Ramos et al., 2008; Díaz-Hernández et al., 2010; Figure 3). This toxic effect may result in cell death and the subsequent detection of tau in the CSF of patients with disorders such as AD (Iqbal et al., 2005; Yamada et al., 2011). Additionally, in affected regions such as the hippocampus, there is an inverse relationship between the numbers of surviving cells and extracellular tangles (Cras et al., 1995; Fukutani et al., 1995). This suggests that degenerating neurons containing fibrillar lesions might release NFT contents into the extracellular environment (Goedert, 1999). Meanwhile, several *in vitro* and *in vivo* studies reported that stimulation of neuronal activity can regulate the physiological secretion of endogenous tau by cortical neurons and enhance the release of pathological tau, a process that is Ca^2+^-dependent and modulated by phosphorylation (Sokolow et al., 2015; Fá et al., 2016; Wang et al., 2017). AMPA receptor stimulation promotes tau release through a Ca^2+^-dependent mechanism and the exocytosis of presynaptic vesicles. AMPA receptor stimulation generates action potentials that increase presynaptic Ca^2+^ concentrations, evoking vesicle release (Schmitz et al., 2009), and this plays a role in Ca^2+^-mediated regulation of neuronal tau release (Pooler et al., 2013). The relationship between neuronal activity and tau release appears to be bidirectional; both extracellular tau and Aβ perpetuate further neuronal tau release through feedback mechanisms (Bright et al., 2015). These results indicate that tau release partially occurs in a neuronal excitability-dependent manner in response to regional changes in the AD brain.

Tau can also be detected in the extracellular space before neurodegeneration, indicating that it can be released through mechanisms other than cell death (Yamada et al., 2011; Barten et al., 2012). Preliminary results demonstrated that tau can be released into the extracellular space via membrane vesicles in the absence of cell death (Simón et al., 2012). Tau secretion likely transpires via the unconventional vesicular- or non-vesicular-mediated secretory pathway since tau lacks an apparent endoplasmic reticulum-targeting sequence, which is necessary in the conventional secretory pathway (Yamada, 2017). Vesicle-mediated secretion might serve as a mechanism to regulate (proteostasis) cellular tau concentrations, maintaining them below a certain threshold level (Simón et al., 2012). Tau can also be transported through membrane vesicles after lysosomal degradation (García-Sierra et al., 2008). Four different mechanisms have been proposed for the unconventional secretion of soluble, cytoplasmic tau: (1) non-vesicular direct translocation from the cytoplasm across the plasma membrane, (2) release via secretory lysosomes that fuse with plasma membranes and release their contents into the extracellular space, (3) microvesicle shedding from the plasma membrane, and (4) vesicle-mediated exosome release (Nickel and Rabouille, 2009; Saman et al., 2012). In the last two scenarios, tau is surrounded by a membrane when it is released into the extracellular space (Nickel and Rabouille, 2009; Chai et al., 2012); Figure 3). It has also been proposed that tau may be released from cells through an exosome-independent pathway that requires heat shock cognate 70, its co-chaperone DnaJ (Hsp40), and synaptosomal-associated protein 23 (Fontaine et al., 2016). Additionally, tau secretion reportedly occurs through membrane vesicles when tau is overexpressed (Simón et al., 2012). Similarly, tau can be secreted in an exosome-dependent manner by Neuro2a cells overexpressing tau (Wang et al., 2017), as well as by microglia (Asai et al., 2015). Another vesicular-mediated mechanism involves large extracellular vesicles called exosomes that are directly shed from cells by plasma membrane budding (Théry et al., 2009; Figure 3). The third mechanism proposed to mediate tau release and spreading involves formation of thin membranous bridges called tunneling nanotubes (TNTs; Rustom et al., 2004). Moreover, cell depolarization was shown to induce the release of a 20-kDa tau fragment from AD synapses (Sokolow et al., 2015). These four mechanisms appear to be temperature dependent and are likely to be less efficient at low temperatures (Nickel and Rabouille, 2009).

Different forms of tau protein might be secreted through different mechanisms or vectors. Endogenous tau released from primary cortical rat neurons under basal conditions is predominantly full length (Pooler et al., 2013), but truncated species have also been identified (Dujardin et al., 2014a). Tau fragments lacking the proline-rich region are either not secreted or are secreted in a manner different from that of the full-length molecule (Pérez et al., 2016). Monomeric and aggregated tau have been detected in CSF, suggesting that they may be released following axonal degeneration and neuronal death (Hampel et al., 2004). However, other studies found that unstimulated human and rodent neurons only secrete C-terminally truncated forms of endogenous tau (Kanmert et al., 2015). Cell culture studies revealed that tau is released via the unconventional secretory pathway and that tau mutations influence the secretion rate, with 4R tau isoforms less abundant than 3R isoforms (Karch et al., 2012). Moreover, exogenously expressed hyperphosphorylated tau secreted by non-neuronal cells is cleaved at its C-terminus (Plouffe et al., 2012; Croft et al., 2017). For instance, tau cleavage at D421 can increase the secretion rate (Katsinelos et al., 2018). Aberrantly phosphorylated tau is secreted more efficiently than non-phosphorylated tau, at least in cultured cell lines (Plouffe et al., 2012; Katsinelos et al., 2018), possibly because abnormal phosphorylation impairs tau’s ability to interact with its partners, therefore altering the protein’s normal physiological properties (Yan et al., 2020). Meanwhile, endogenous tau is reportedly released either free and in a full-length, dephosphorylated form (Pooler et al., 2013) or as N-terminally truncated fragments (Bright et al., 2015; Kanmert et al., 2015). A small subset of tau released under these conditions is inside plasma membrane-derived vesicles called ectosomes (Dujardin et al., 2014a). Tau is also released from cells in association with exosomes, particularly when it is exogenously expressed or in a highly phosphorylated and misfolded state (Plouffe et al., 2012; Saman et al., 2012). One study suggested that tau hyperphosphorylation in AD may induce a vicious circle that amplifies its secretion (Plouffe et al., 2012). In this case, tau hyperphosphorylation would enhance its secretion, which would subsequently increase the level of dephosphorylated tau in the extracellular space. Dephosphorylated extracellular tau would then induce an increase in intracellular Ca^2+^ concentrations, which has been linked with elevated tau hyperphosphorylation (Díaz-Hernández et al., 2010). This vicious circle then promotes tau pathology propagation in the brain and CSF accumulation (Hampel et al., 2010; Plouffe et al., 2012). Golgi dynamics were also proposed to modulate tau secretion from both HeLa cells and primary cortical neurons (Mohamed et al., 2017). Tau cleavage and hyperphosphorylation increase its secretion from HeLa cells. Mitochondrial damage might reduce tau secretion (Shafiei et al., 2017), whereas impaired lysosomal function may increase it (Mohamed et al., 2014). Pathological tau in animal models appears to be more localized to synapses compared to non-pathological tau (Sahara et al., 2014), and synaptosomes isolated from human AD brains were shown to contain more phosphorylated and aggregated tau than those isolated from healthy controls (Tai et al., 2014). In summary, different tau species and isoforms including mutated (Hampel et al., 2004), hyperphosphorylated (Plouffe et al., 2012; Bright et al., 2015), and truncated forms of tau appear to be released via distinct mechanisms (Plouffe et al., 2012).

## The Spread of Pathological Tau Protein

**FIGURE 4:**
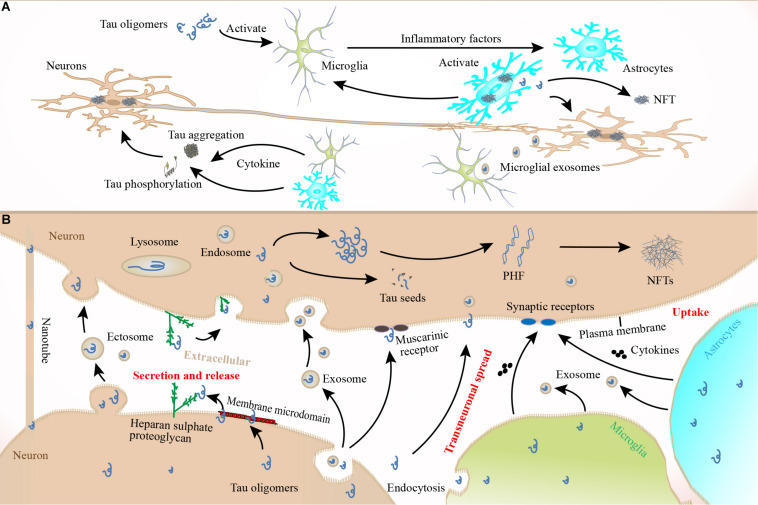
Tau pathology spread. Once released into the extracellular space, tau can be transported to connected cells via exosomes or ectosomes. Tau can also be transported to other cells by binding to muscarinic (M1, M3) receptors or synaptic receptors or via special structures such as membrane microdomains and HSPGs. There may also be nanotubes between cells, which can also spread tau oligomers. The plasma membrane may play a role in tau transmission through endocytosis-mediated uptake of tau oligomers. Microglia can also phagocytose secreted tau oligomers that are then spread to healthy neurons in exosomes, and astrocytes may be involved in this process. Both microglia and astrocytes can secrete cytokines that act on connected neurons, which further accelerates tau pathology. However, the specific mechanisms underlying the roles of microglia and astrocytes in tau pathology spread remain unclear. When tau oligomers or monomers are released by lysosome or ingested by recipient cells, they will misfold and form toxic seeds, PHFs, or NFTs. Once the recipient cells spread the pathological tau fragments to other cells, this perpetuates a vicious cycle that exacerbates AD.

Although the specific routes and mechanisms underlying the spread of pathological tau remain unclear (Braak and Braak, 1991), the propagation of cytosolic tau to connected neurons consists of at least four phases. First, tau must be secreted or released from donor neurons; second, it must undergo aggregation before or after being released; third, tau must be taken up into recipient neurons; and fourth, tau aggregation must be induced in recipient cells (Kanmert et al., 2015). After the release and secretion of tau to the extracellular space following cell death (Simón et al., 2012), stimulation of neuronal activity (Pooler et al., 2013), or other mechanisms (e.g., associated with vesicles, secretory lysosomes, or microvesicle shedding from the plasma membrane) (Nickel and Rabouille, 2009; Saman et al., 2012), pathological tau oligomers, monomers, or aggregates must enter other cells via endocytosis. Subsequently, pathological tau seeds might be degraded, resecreted, or mediate the misfolding of wild-type tau molecules in recipient cells (Wu et al., 2013). Recipient cells appear to favor the uptake of short, low-molecular-weight, tau fibrils over monomers and larger fibrils (Wu et al., 2013). Consequently, the potencies of various tau aggregate species on cellular propagation may be different (Frost et al., 2009). For example, tau uptake is closely related to both the size and conformation of tau aggregates (Kanmert et al., 2015). Intracellular tau accumulation is dependent on the isoform composition of the extracellular tau oligomers (Swanson et al., 2017). However, tau aggregate uptake is not neuron specific; cell-to-cell transfer also occurs between glial cells and neurons. Tau aggregates can transfer between connected cells, induce templated misfolding, and be internalized from the extracellular space by a neighboring cell, which facilitates tauopathy propagation across different brain regions in a prion-like fashion (Jucker and Walker, 2013). Tau oligomers can be internalized by dynamin-dependent bulk endocytosis and are then transported through the endolysosomal pathway in recipient cells (Wu et al., 2013). One study demonstrated that neuronal uptake of α-synuclein and tau aggregate seeds can occur through macropinocytosis, a form of fluid-phase bulk endocytosis that represents the most likely mechanism for tau uptake. This process begins when aggregated proteins bind to HSPGs, a family of core proteins with cell-surface glycosaminoglycan polysaccharides. Interestingly, the internalization process is only initiated by aggregated species, not by monomeric tau (Holmes et al., 2013). Another study also suggested that HSPGs can mediate exosome internalization (Christianson et al., 2013; Fuster-Matanzo et al., 2018). Before internalization via micropinocytosis, tau binds to plasma membrane HSPGs, which promotes membrane rearrangement before endocytosis (Holmes et al., 2013). Tau binding to HSPGs seems to be essential for internalization, and the 6-O-sulfation pattern on heparan sulfate sidechains is an important determinant for tau binding (Rauch et al., 2018; Figure 4). However, heparan-like glycosaminoglycan (GAG) mimetics can hide tau’s HSPG binding site, which reduces cell-surface tau oligomer binding, uptake, and seeding (Holmes et al., 2013). HSPG-dependent macropinocytosis is instigated by small protein aggregates, and tau trimers were shown to be the smallest size able to initiate this mechanism (Mirbaha et al., 2015; Rauch et al., 2018). The results of a recent study also supported the hypothesis that different tau species could be internalized through different cellular mechanisms (Evans et al., 2018). For example, tau monomers and small oligomers are preferentially taken up by macropinocytosis, while dynamin-dependent endocytosis is the preferred route for larger aggregates. HSPGs and macropinocytosis may also play a role in the uptake of whole exosomes (Christianson et al., 2013), although exosomes are internalized because of this pathway’s non-specificity. In short, endocytosis and/or pinocytosis might be favored over direct fusion to the plasma membrane as an exosome internalization route (Tian T. et al., 2013; Polanco et al., 2018). During this process, vesicles can be endocytosed by neighboring cells, which might be involved in the propagation of misfolded or aggregated tau proteins in different neurodegenerative disorders (Goedert et al., 2010; Guo and Lee, 2011). Meanwhile, micropinocytosis might be critical for pathological tau uptake both *in vitro* and *in vivo* (Holmes et al., 2013; Brunello et al., 2016), as well as the uptake of other pathologically misfolded proteins including α-synuclein, TDP-43 (Zeineddine et al., 2015), and PrP (Hooper, 2011). Tau aggregates are reportedly internalized into primary neurons, where they are trafficked anterogradely and retrogradely along axons, and then spread to connected cells (Takeda et al., 2015; Wu et al., 2016; Wang et al., 2017), thereby propagating tau pathology (Calafate et al., 2015; Wu et al., 2016; Nobuhara et al., 2017). They can also seed the aggregation of native monomers, thereby initiating more aggregates that are then released and spread to neighboring cells (Kundel et al., 2018; Brunello et al., 2020; Figure 4). Recent reports found that inoculation of preformed tau fibrils into tau transgenic mice quickly induced an AD-like NFT pathology in connected brain regions (Clavaguera et al., 2013; Ahmed et al., 2014; Boluda et al., 2015). Moreover, misfolded tau proteins spread through anatomically connected neurons, presumably via trans-synaptic tau aggregate transmission (Harris et al., 2012; Liu et al., 2012). However, preformed tau aggregates can also spread by means other than synaptic connections, suggesting the existence of alternative (non-synaptic) propagation pathways (de Calignon et al., 2012; Peeraer et al., 2015).

How neurons release tau that is then transmitted to recipient neurons to instigate tau propagation remains unclear. Although synaptic connections can facilitate tau’s transcellular spread, other cellular uptake routes cannot be excluded. Once internalized, tau can be located in both early and late endosomes, which links these tau species to lysosomal vesicles in a retrograde axonal pathway and provides further evidence for a transsynaptic route of transmission (Frost et al., 2009; Wu et al., 2013). Extracellular vesicles play a major role in cellular communication and the transport of pathogenic proteins related to AD (Vingtdeux et al., 2012). Notably, exosomal tau protein levels are elevated in prodromal AD (Fiandaca et al., 2015; Gibbons et al., 2019). Microglia also contribute to tauopathy progression via exosome secretion, such that microglia depletion and inhibition of exosome synthesis can dramatically suppress tau propagation *in vitro* and *in vivo* (Asai et al., 2015). Additionally, astrocytes can internalize both fibrillar and monomeric tau, implying that these cells are also be involved in tau pathology spread (Martini-Stoica et al., 2018; Perea et al., 2019). However, one study found that oligomers and short fibrils that bind to the membrane can be internalized by neuronal cells via a receptor-independent mechanism, while tau monomers, long fibrils, and long filaments cannot (Wu et al., 2013). Thus, these structures (exosomes, ectosomes, or TNTs) mediate neuron-to-neuron transfer of pathological tau protein assemblages, which is considered a fast manner of tau spread that is prion like (Abounit et al., 2016; Tardivel et al., 2016); Figure 4). Tau protein modifications can also affect the spread of tau pathology. Tau hyperphosphorylation can enhance spread, while partial dephosphorylation slows it (Alonso et al., 1996). This observation indicates that tau hyperphosphorylation may be a potential target to prevent tau pathology progression in AD and other tauopathies (Hu et al., 2016). Tau fibrils can propagate by incorporating unphosphorylated tau monomers that undergo conformational changes and are then hyperphosphorylated (Goedert et al., 2017). The extracellular domain of the amyloid precursor protein might be involved in tau fibril uptake into cells (Takahashi et al., 2015). It is well known that increasing Aβ42 oligomerization can activate protein kinases (including GSK-3β) that phosphorylate tau (Guo et al., 2013). Aβ aggregation can promote tau hyperphosphorylation, suggesting that Aβ might accelerate the spread of tau pathology (Pérez et al., 2018).

## Toxicity Associated With the Spread of Tau Pathology

Tau is normally enriched on MTs within axons. In tauopathies, tau is hyperphosphorylated and accumulates in the somatodendritic compartment of brain cells, which is one of the pathological hallmarks of AD (Lee et al., 2001). Tau aggregates into insoluble filaments, forming NFTs (Morris et al., 2011) that are associated with cognitive deficits (Braak and Braak, 1991; Bierer et al., 1995; Plouffe et al., 2012). Tau phosphorylation, mislocalization, and conformational changes can alter Ca^2+^ homeostasis, induce dendritic spine loss, impair organelle trafficking (particularly mitochondria), and lead to cell death (Dixit et al., 2008; Zempel et al., 2010; Li et al., 2011; Spires-Jones et al., 2011). These phenotypes represent a pretangle stage, which is widely recognized as an early event in the pathological process of AD (Eckermann et al., 2007; Braak and Del Tredici, 2012). Three hypotheses have been proposed to underlie tau-mediated toxicity. First, insoluble NFTs may be toxic and lead to neuron death and cognitive dysfunction in AD; second, soluble species of misfolded, hyperphosphorylated tau may become toxic when they accumulate in inappropriate cellular compartments, whereas NFTs exert a protective effect by serving as a sink for these toxic species; and in the third view, soluble forms of pathological tau and insoluble NFTs are both toxic to cells in various ways and time scales (Kopeikina et al., 2012). We favor the third view. Neuronal transport disruption is considered an important form of tau toxicity, which is an early phenomenon and underlying cause of neurodegenerative conditions including AD (Lin and Beal, 2006; Morfini et al., 2009; Wang and Schwarz, 2009; Querfurth and LaFerla, 2010; Kopeikina et al., 2012). Recent studies have found that soluble tau species are related to synaptic or neuronal dysfunction (Berger et al., 2007; Polydoro et al., 2009; Hoover et al., 2010; Sydow et al., 2011), with results indicating that tau oligomers—but not monomers or fibrils—act as aggregation seeds in the brains of wild-type mice, leading to mitochondrial dysfunction, synaptic deficits, and memory impairment (Usenovic et al., 2015). Based on these results, the interneuronal spread of these soluble tau species might be involved in the spread of AD pathology through the brain (Braak and Del Tredici, 2012; de Calignon et al., 2012; Clavaguera et al., 2013). Furthermore, neurons can endocytose low-molecular-weight misfolded tau species (but not monomeric tau) that are transported anterogradely and retrogradely, resulting in endogenous tau pathology *in vivo*. However, they cannot endocytose fibrillar tau or brain-derived filamentous tau (Wu et al., 2013). This evidence strongly indicates that tau toxicity may be mediated by the cell-to-cell spread of trimeric and larger oligomeric forms in certain brain regions by endocytosis (Tian H. et al., 2013). Extracellular tau is neurotoxic (Gómez-Ramos et al., 2006) and contributes to the spread of AD pathology. When the extracellular level of free tau exceeds that inside the cell and reaches a critical concentration (Reynolds et al., 2005), tau protein will self-aggregate and induce extracellular toxicity. Paired-helical filament (PHF)-tau is less toxic than free tau. When tau interacts with muscarinic receptors, intracellular Ca^2+^ levels increase due to Ca^2+^ release from intracellular stores (Gómez-Ramos et al., 2006). Tau seeds can also cause toxicity and cell death via Ca^2+^ dysregulation (Querfurth and LaFerla, 2010; Tian H. et al., 2013; Hallinan et al., 2019); altered intracellular Ca^2+^ homeostasis results in tau phosphorylation, which is related to tau pathology progression in AD (Delacourte et al., 1999). Tau phosphorylation will increase tau detachment from MTs (Avila et al., 2004), increasing free tau levels. Free tau is then released during neurodegeneration or following cell death and binds to muscarinic receptors on surrounding cells, thereby inducing muscarinic toxicity and aggravating tau toxicity and transmission (Gómez-Ramos et al., 2008). In addition, different tau forms or isomers secreted into the extracellular space via different mechanisms may play varied roles in AD pathogenesis. Certain forms of tau released through cell death or neuroactive stimulation may be non-toxic, while misfolded tau fragments or seeds (induced by Aβ, kinases, and hydrolases) exert toxic effects on the extracellular space. Secreted vesicles containing tau protein inhibit tau binding to muscarinic receptors, thus reducing neurotoxicity.

The neurodegenerative consequences of tau hyperphos- phorylation include axonal transport impairment (Ittner et al., 2008), tau relocalization to the somatodendritic compartment, and synaptic loss (Di et al., 2016). Synaptic dysfunction can occur both presynaptically, where it can interfere with the transport of phosphorylated tau via synaptic vesicles (Zhou et al., 2017), and postsynaptically via the downregulation of AMPA receptors (Hoover et al., 2010). In the context of the prion hypothesis, tau assemblies that enter the cytoplasm can seed native monomer aggregation, and these species can be released and spread to neighboring cells (Clavaguera et al., 2013; Kundel et al., 2018). Under pathological conditions, aberrant posttranslational modifications such as hyperphosphorylation, truncation, deamidation, and others (Avila et al., 2004) can induce tau detachment from MTs and promote their accumulation in a free form. When neurons degenerate and die, this free tau enters the extracellular space, where it is free to diffuse in every direction (Guo and Lee, 2011; Hu et al., 2016). This is in line with the observation that neuron loss is progressive within brain areas affected by degeneration, and tau species can seed misfolding in human brains without tangles (DeVos et al., 2018). This implies that tau seeds are released from intact neurons prior to neuronal death (Pickett et al., 2017). Another possible explanation of tau pathology patterns in the brains of AD patients is that extracellular NFTs or other substances released by degenerating neurons accumulate in the extracellular space and damage nearby cells (Avila, 2006). These toxic compounds can act like extracellular Aβ peptides (Gómez-Ramos et al., 2006). Thus, both soluble tau species and insoluble NFTs may contribute to the spread of tau toxicity.

## Conclusion and Prospects

The mechanism involved in the transcellular propagation of tau in neurodegeneration is still unclear. Studies on the molecular mechanisms underlying the release, propagation, and uptake of tau are needed; improving the ability to detect secreted tau species is also important. Future research should focus on reducing the secretion and generation of extracellular tau in a soluble or aggregated form and inhibiting cell uptake. However, tau aggregate species are diverse, and it is currently unclear if certain species prefer certain secretion pathways. It will be important to clarify whether the physiologic secretion of non-pathological tau from neurons occurs via the same or overlapping mechanisms as those for the pathologic forms. Moreover, both forms of tau secretion are connected to neuronal activity, so blocking synapse-mediated tau propagation and boosting the clearance of tau aggregates that are internalized at the synapse are equally important (Calafate et al., 2015). More attention should also be given to glial cells and the glymphatic system, which might play a role in clearing and propagating pathological protein aggregates. Inhibition of donor cell release and recipient cell uptake are also novel therapeutic directions worthy of consideration. For example, it might be possible to block exosome secretion pathways and apply tau antibodies that can act on pathological tau fragments in the extracellular space and inhibit tau aggregation on membranous structures. Recent studies have shown that anti-tau antibodies can reduce tau hyperphosphorylation and aggregation in the transgenic mouse brain (Yanamandra et al., 2013; Sankaranarayanan et al., 2015). In addition, preventing tau from binding to HSPGs precludes recombinant tau fibrils from inducing intracellular aggregation and blocks transcellular aggregate propagation. *In vivo*, the heparin mimetic F6 prevents neuronal uptake of tau fibrils injected stereotactically (Holmes et al., 2013). Moreover, microglia disseminate tau via exosome secretion, and hampering exosome synthesis significantly reduces tau propagation *in vitro* and *in vivo*, which implies that exosomes and microglia contribute to tauopathy progression. It also suggests that targeting the exosome secretion pathway could be therapeutically useful. Depleting microglia dramatically suppressed tau propagation and reduced excitability in the dentate gyrus in a mouse model of AD (Asai et al., 2015). In summary, tau release mechanics can be explored to develop new treatments for AD and other tauopathies.

## Author Contributions

HZ wrote the manuscript. YC and LM assisted in the manuscript writing. YW and HL assisted in ideas and modification of the manuscript. All authors contributed to the article and approved the submitted version.

## Conflict of Interest

The authors declare that the research was conducted in the absence of any commercial or financial relationships that could be construed as a potential conflict of interest.

## Publisher’s Note

All claims expressed in this article are solely those of the authors and do not necessarily represent those of their affiliated organizations, or those of the publisher, the editors and the reviewers. Any product that may be evaluated in this article, or claim that may be made by its manufacturer, is not guaranteed or endorsed by the publisher.
